# The Synergy of Aging and LPS Exposure in a Mouse Model of Parkinson’s Disease

**DOI:** 10.14336/AD.2017.1028

**Published:** 2018-10-01

**Authors:** Yong-Fei Zhao, Qiong-Zhang, Jian-Feng Zhang, Zhi-Yin Lou, Hen-Bing Zu, Zi-Gao Wang, Wei-Cheng Zeng, Kai-Yao, Bao-Guo Xiao

**Affiliations:** ^1^Department of Neurology, Jinshan Hospital, Fudan University, Shanghai, China; ^2^Institute of Neurology, Huashan Hospital, Institutes of Brain Science and State Key Laboratory of Medical Neurobiology, Fudan University, Shanghai, China; ^3^Department of Neurology, Xinhua Hospital, Medical College, Shanghai Jiaotong University, Shanghai, China

**Keywords:** Aging, Parkinson’s disease, Lipopolysaccharides, neuroinflammation, oxidative stress

## Abstract

Aging is an inevitable physiological challenge occurring in organisms over time, and is also the most important risk factor of neurodegenerative diseases. In this study, we observed cellular and molecular changes of different age mice and LPS-induced Parkinson disease (PD) model. The results showed that behavioral performance and dopaminergic (DA) neurons were declined, accompanied by increased expression of pro-inflammatory factors (TLR2, p-NF-kB-p65, IL-1β and TNF-α), as well as pro-oxidative stress factor gp91phox in aged mice compared with young mice. Aging exaggerated inflammatory M1 microglia, and destroyed the balance between oxidation and anti-oxidation. The intranasal LPS instillation induced PD model in both young and aged mice. The poor behavioral performance and the loss of DA neurons as well as TLR2, p-NF-kB-p65, IL-1β, TNF-α, iNOS and gp91phox were further aggravated in LPS-aged mice. Interestingly, the expression of Nrf2 and HO-1 was up-regulated by LPS only in young LPS-PD mice, but not in aged mice. The results indicate that the synergy of aging process and LPS exposure may prominently aggravate the DA neurons loss caused by more serious neuroinflammation and oxidative stress in the brain.

Aging is an inevitable physiological challenge occurring in organisms over time, and is the most important risk factor of neurodegenerative diseases [1]. Age-related diseases and disability has increasingly become a pressing problem along with the average life expectancy extending. The prevalence of Parkinson’s disease (PD) increases with increasing age [2, 3]. PD is the second most common neurodegenerative disease worldwide and is deeply concerned because of its irreversible course and the lack of effective treatments [4, 5]. Therefore, PD has become a serious public health problem [4, 6].

The precise etiology of PD is still unclear [4, 7]. Loss of dopaminergic (DA) neurons in the substantia nigra (SN) is the major pathologic phenomenon in PD [8]. It is postulated that multiple events, such as protein aggregation, impairment of the ubiquitin proteasome pathway, mitochondrial dysfunction, neuroinflammation, and oxidative stress, may be involved in the pathogenesis of PD [6]. Aging is the primary risk factors for PD, which is characterized by a tenfold accelerated loss of nigral DA neurons[9]. In PD, protein homeostasis become gradually incapacitated with age, leading to the onset of neurodegenerative diseases [10]. Human monocytes from elderly donors showed reduced phagocytic activity of extracellular α-synuclein [11]. In addition to the progressive accumulation of molecular damage inside the cells, aging is associated with an overall decrease in proteasome activity, impaired autophagy, and mitochondrial dysfunction [12-17]. It was reported that lysosomal degradation was significantly impaired in aging models, while anti-aging treatment reduced the impairment [18]. Postmitotic neurons are expected to be more susceptible to the cumulative effects of aging as they do not self-renew by dividing. Continuous exposure to a pro-inflammatory microenvironment drives exaggerated changes in the production and release of inflammatory mediators that interact with age to impair functional capacity of the substantia nigra pars compacta (SNC) and the locus coeruleus (LC) [19]. However, the pathogenesis and clinical manifestations of PD should be the result of complex interactions of aging with other susceptibility [20-22]. Although PD and age are highly interconnected, the field still lacks detailed insights into the specific interplay between different pathways and processes that may ultimately influence PD pathogenesis [23]. Therefore, understanding PD pathogenesis and risk factors associated with age may contribute to the development of therapies that are able to slow or prevent the progression of this neurodegenerative disorder.

The current knowledge regarding pathogenetic mechanisms of PD has obtained from studies in animal models of PD, generated by toxins or genetic manipulation. However, none of the current models fully reproduces the complex pathogenicity of PD, particularly in relation to the chronic progressive process of neurodegeneration [24]. Neuroinflammation is an important feature in the pathogenesis and progression of neurodegenerative diseases such as PD [1, 25]. Brains from patients with neurodegenerative diseases are characterized by astrocytosis, activation of microglia and increase of pro-inflammatory cytokines [21, 25, 26]. It has been well documented that lipopolysaccharide (LPS) induced the loss of DA neurons in SN mediated, in part, by pro-inflammatory cytokines released mainly from microglia in the brain. Previous studies also confirmed the co-existence and complex interplay between inflammatory response and oxidative stress, contributing equally to the process of DA neuronal degeneration in PD [27].

Epidemiological studies show that PD is associated with age and increases with age. The precise pathogenesis of PD remains unclear, and may be associated with a variety of factors. But there is no clear understanding why PD occurs in an elderly population. In this study, we utilized LPS-induced PD model in mice with different age to explore the impact of aging to the development of PD. We also further investigated the impact of synergy between aging and LPS exposure to the neuroinflammation and oxidative stress, especially the relation of double hit to the loss of DA neurons.

## MATERIALS AND METHODS

### Experimental PD model and design of treatment

Female C57BL/6 young mice (10-12 weeks old and 20-22g weight) and female C57BL/6 aged mice (15 months old and 33-38g weight) were purchased from shanghai SLAC laboratory animal Co. Ltd. (Shanghai, China). Mice were kept under pathogen-free conditions for 12-h light/12-h dark cycle in a temperature control system (25 ± 2 °C), with free access to food and water for 1 week prior to experimental manipulation. All experiments were approved by the Ethics Committee of Fudan University, Shanghai, China (Approval No. 20171542A493), and were conducted in accordance with the guidelines of the International Council for Laboratory Animal Science.

LPS (1 mg/ml; Sigma-Aldrich, USA) was dissolved in normal saline (NS) just before bilateral nasal administration. After a slight anesthesia with ether, mice were held by the neck and were laid upside down with a finger under the neck to limit liquid flow down the trachea.

A total of 16 aged mice were randomly divided into 2 groups (n=8 per group): aged LPS-PD group and aged control group. In addition, a total of 16 young mice were also randomly divided into 2 groups (n=8 per group): young LPS-PD group and young control group. In LPS-PD model, LPS (10 µl/each side) was equally instilled into bilateral nasal cavity of aged and young mice every other day for continuous 60 days. Meanwhile, the equal NS was slowly instilled into bilateral nasal cavity of aged and young control mice in the same way [28].

### Behavior analysis

Behavior analysis was performed by adhesive remove test (ART) and pole test to observe the balance and coordination function of automatic movement. Behavior training and learning once daily were started 2 days before test. Behavior analysis was performed once daily, lasting for 3 days.

*ART*: At the end of 60^th^ days, ART was performed with the method described by Bouet *et al* [29]. Briefly, mice were put into standard cages 1 min before starting the experiment to allow habituation to the new environs. Prior to test, the training trails were gently performed once daily by applying the two adhesive dots (0.6 cm in diameter) with equal pressure on each forelimb plantar surface, which decreased their anxious responses during formal experiment. The time of removing the dot from each forelimb was recorded, with a maximum time of 120s.

*Pole test:* Pole test was also conducted to measure motor balance and coordination of mice as described previously [30] with minor modification. In the study, mice were placed on the top of a vertical rough-surfaced pole (diameter: 12 mm; height: 80cm) and allowed to descend to the base of the pole. The time descending the pole was recorded with a maximum duration of 120s. Even if the mouse dropped part way and fell the rest of the way, the behavior was scored until it descended to the floor. When mice were not able to turn downward instead of dropping from the pole, latency was taken as 120s (default value). ART and Pole tests were conducted by two observers under double-blind condition.

### Brain protein extraction

After the behavior test, mice were euthanized by intraperitoneal injection of 10% chloral hydrate (3 ml/kg), and then perfused with NS. Brains (n=4) were removed immediately and stored at -80 ? for protein extraction. Brains were homogenized in the lysis buffer (RIPA lysis buffer: PMSF = 100: 1) on the ice for 20 min. Protein concentrations were determined by the Enhanced BCA Protein Assay Kit according to the manufacturer’s instructions.

### Western blot

Equal amounts of brain protein (30 µg) were separated on a sodium dodecyl sulfate polyacrylamide gel electrophoresis (SDS-PAGE) and transferred onto nitrocellulose membranes. After blocking with 5% non-fat dry milk at room temperature (RT) for 1 h, the membranes were then incubated at 4 °C for 24 h with corresponding primary antibodies against tyrosine hydroxylase (TH) (MAB 318, 1: 1000 dilution, Millipore), TLR2 (ab108998, 1:1000, abcam), p-NF-kB-p65 (cst 3033, 1:1000, Cell Signal Technology), arginase 1 (arg-1)(ab124917, 1:500, abcam), iNOS (ab178945, 1:300, abcam), Nrf2 (ab92946, 1:1000, abcam), HO-1 (ab68477, 1:2000, abcam), gp91Phox (ab180642, 1:1000, abcam), glyceraldehyde-3-phosphate dehydrogenase (ab181603, GAPDH1: 2000, abcam). After washing with TBST, the immunoblots were incubated with horseradish peroxidase (HRP)-conjugated secondary antibodies at RT for 1 h. After washing with TBST again, the immunoblots were applied with an enhanced chemiluminescence (ECL) reagents (Millipore, USA), and the ECL was visualized under ECL system (GE Healthcare Life Sciences), followed by imaging and quantification of protein bands using Bio-Rad Quantity One 1-D software. GAPDH was used as a loading control.

### Cytokine ELISA

The concentrations of cytokine IL-1β (88-7324-88) and TNF-α (88-7013-88) in the brain tissue extract were detected with the ELISA kits (ELISA Development Kit, Peprotech, USA) according to manufacturer’s instructions. The results were expressed as pg/10mg protein.

### Immunohistochemistry

Mice were anesthetized and perfused with PBS and 4% buffered paraformaldehyde. Brain slices (10 μm) were blocked with 3% bovine serum and permeabilized with 0.3% Triton X-100 in 1% BSA-PBS for 30min, and incubated at 4 °C overnight with antibodies against TH (MAB318, 1: 500, Millipore), TLR2 (ab108998, 1:100, abcam), p-NF-КB-p65 (cst 3033, 1:100, Cell Signal Technology), arg-1 (ab124917, 1:50, abcam), iNOS (ab178945, 1:50, abcam), Nrf2 (ab92946, 1:50, abcam), HO-1 (ab68477, 1:50, abcam), gp91phox (ab180642, 1:50, abcam), CD11b (14-0112-85, 1:100, eBioscience). The slices were then incubated with corresponding secondary antibodies (1:1000, Cell Signaling Technology) at RT for 1h. Hoechst 33342 (Sigma) was used to identify the nuclei. The control sections were run following identical protocol, but omitting the primary antibodies.

After the immunohistochemistry staining, sections were covered with slipped 50% glycerol, and blindly examined. According to the mouse brain atlas, the thickness of the SN was about 1.5 mm. We cut 150 pieces in total from anterior to posterior of the SN, and then chose six sections from each 25 pieces. Each section in the different groups was at the same level of the SN. TH-immunoreactive (TH-ir) neurons were stereologically counted from anterior to posterior of the SN.

Quantitative analysis of the histological data (the expression of TLR2, p-NF-кB-p65, arg-1, iNOS, Nrf2, HO-1 and gp91phox on CD11b^+^ microglia) was carried out based on number of double positive cells/mm^2^ by Image-pro Plus software.

### Statistical analysis

All data were obtained from at least three independent experiments and were expressed as means ± standard error measurement (SEM). Multiple comparisons were analyzed by one-way ANOVA. P<0.05 was considered to be statistically significant. All statistical analyzes and graphs were performed or generated with GraphPad Prism v5.0 (GraphPad Prism Software, Inc, san diego, calif., USA).

## RESULTS

### Behavioral alterations

**Figure 1.**
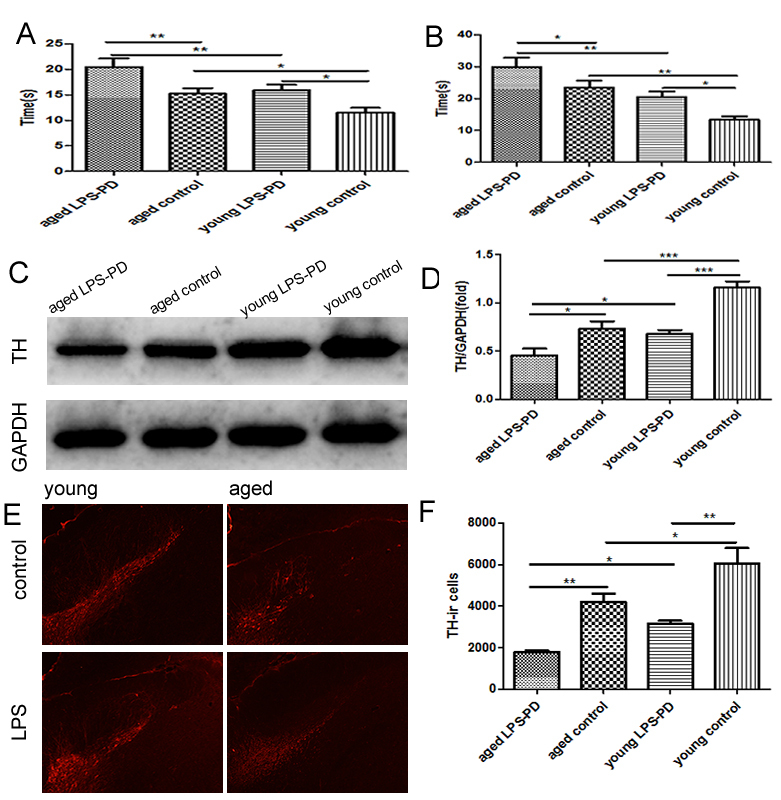
Behavioral alterations and TH expression and TH neurons in young and aged mice stimulated with or without LPS 10-12-week B6 mice were given with saline (young control group, n=8) or intranasal LPS (young LPS-PD group, n=8) for 2 months. 15-month B6 mice were given with saline (aged control group, n=8) or intranasal LPS (aged LPS-PD group, n=8) for 2 months. **A)** adhesive remove test (ART), and **B)** pole test. TH expression in brain was assayed by Western blot. **C)** Representative bands of TH immunostaining. **D)** quantitative data of TH immunostaining. TH neurons in SN of brain was assayed by immunohistochemistry. **E)** Representative images of TH immunohistochemistry. **F)** quantitative data of TH positive neurons. Quantitative results of behavioral alterations are mean ± SD of 8 mice in each group, and the quantitative results of TH expression are mean ± SEM of 4 mice in each group. *p<0.05, **p<0.01, ***p<0.001.

Pole test showed that the average time in aged LPS-PD group (29.96±2.88s)was significantly extended compared with aged control group (23.46±2.20s, p<0.01)and young LPS-PD group (20.50±1.73s, p<0.01). Meanwhile, the average time in young LPS-PD group and aged control group was significantly extended compared with young control group (13.38±1.05s, p<0.05) respectively (Fig. 1A).

**Figure 2.**
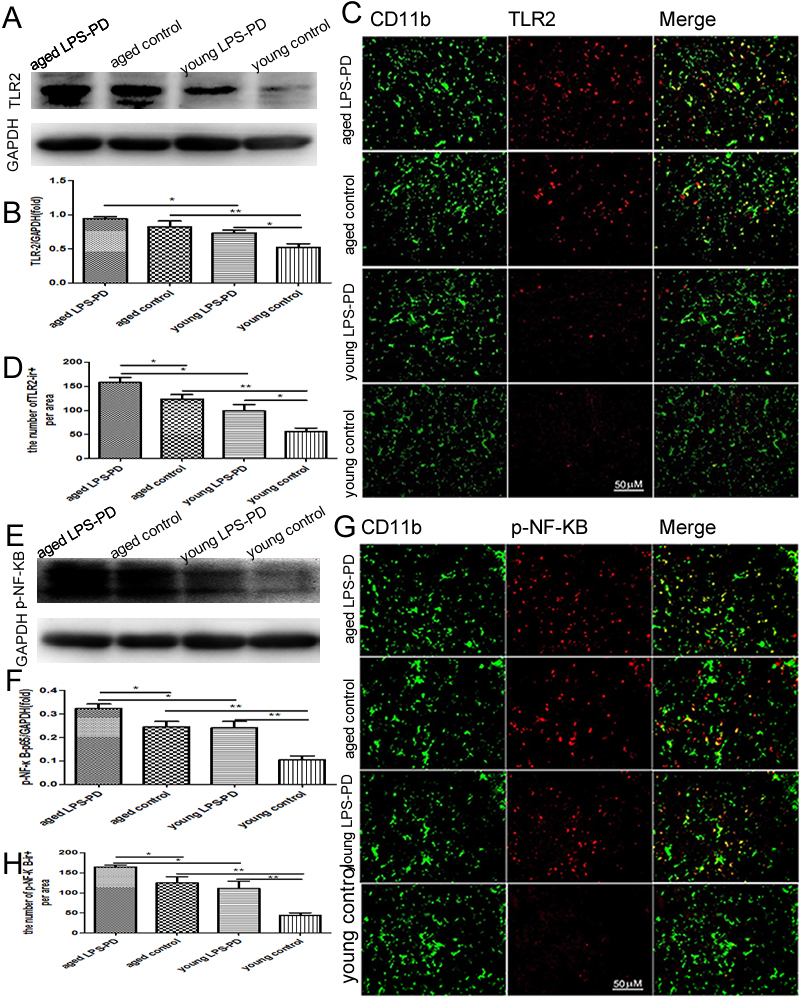
TLR2/p-NF-κB/p65 expression and TLR2/p-NF-κB/p65 microglia in brain of young and aged mice stimulated with or without LPS Mice were dropped into the bilateral nasal cavity with saline or LPS in young or aged mice for 2 months. TLR2/p-NF-κB/p65 expression in brain was assayed by Western blot. **A)** Representative bands of TLR2 immunostaining. **B)** Quantitative data of TLR2 immunostaining. TLR2/p-NF-κB/p65 positive microglia in brain was assayed by double immunohistochemistry of TLR2/p-NF-κB/p65 and CD11b. **C)** Representative images of TLR2 expression on CD11b^+^ microglia. **D)** Quantitative data of double positive cells. **E)** Representative bands of p-NF-κB/p65 immunostaining. **F)** Quantitative data of p-NF-κB/p65 immunostaining. **G)** Representative images of p-NF-κB/p65 expression on CD11b^+^ microglia. **H)** Quantitative data of double positive cells. Quantitative results are mean ± SEM of 4 mice in each group. *p<0.05, **p<0.01.

ART behavioral experiment showed that the average time in aged LPS-PD group (20.47±1.66s) was significantly delayed compared with aged control group (15.22±1.09s, p<0.05) and young LPS-PD group (15.88±1.14s, p<0.01). At the same time, the average time in young LPS-PD group and aged control group was dramatically delayed compared with young control group (11.50±0.97s,p<0.05 and p<0.01 respectively) (Fig. 1B). The results suggest that the dysfunction of balance and coordination in the spontaneous movement of LPS-PD mice retarded with age, and that LPS-PD can be induced with intranasal instillation in both young mice and aged mice.

### The loss of DA neurons was increased with aging and accelerated with LPS

The expression of TH was decreased in aged control group (0.73±0.08) and aged LPS-PD group (0.46±0.07) as compared with young control group (1.16±0.07,p<0.001) and young LPS-PD group (0.68±0.04, p<0.05), which indicated that there had the relationship between the loss of DA neurons and aging (Fig. 1D).

The DA neurons in SN were gradually decreased along with aging (p<0.05), and the loss was accelerated in both young and aged mice attached with intranasal LPS instillation (p<0.01 respectively) (Fig. 1F). The symptoms of PD will emerge when the loss of DA neurons reach a certain threshold.

### TLR2-NF-kB was upregulated with aging and further increased with LPS

Toll-like receptors (TLRs) are expressed in brain microglia, and recognize pathogen- or damage-associated molecules, leading to inflammatory release and toxic molecules, which contribute to neuroinflammation and neurodegeneration associated with PD. As shown in Fig. 2A and 2B, the expression of TLR2 in the brain of young mice (0.52±0.05) was relatively low compared with that of aged mice (0.82±0.09, p<0.01). The intranasal LPS instillation induced the expression of TLR2 in young LPS-PD mice (0.73±0.04, p<0.05), but did not stimulate significant increase of TLR2 expression in aged LPS-PD mice (0.94±0.03, p>0.05).

The results of double immunofluorescence demonstrated that TLR2 was expressed in CD11b positive microglia (Fig. 2C). The intensity of TLR2 expression in different groups was consistent with the results from western blot (Fig. 2D).

NF-kB-mediated cell signaling is one of the common pathways engaged in the inflammatory response with respect to TLR activation. The expression of p-NF-kB/p65 in the brain of young mice (0.10±0.02) was relatively low compared with that of aged mice (0.24±0.02, p<0.01) (Fig. 2F). The intranasal LPS instillation significantly induced the expression of p-NF-kB/p65 in young LPS-PD mice (0.24±0.03, p<0.01) and in aged LPS-PD mice (0.32±0.02, p<0.05) (Fig. 2F). Compared with aged control mice, LPS further stimulated the expression of p-NF-kB/p65 in aged LPS-PD mice (Fig. 2F, p<0.05).

The results of double immunofluorescence demonstrated that p-NF-КB-p65 was expressed in CD11b positive microglia (Fig. 2G). The intensity of p-NF-КB-p65 was consistent with the results from western blot (Fig. 2H).

**Figure 3.**
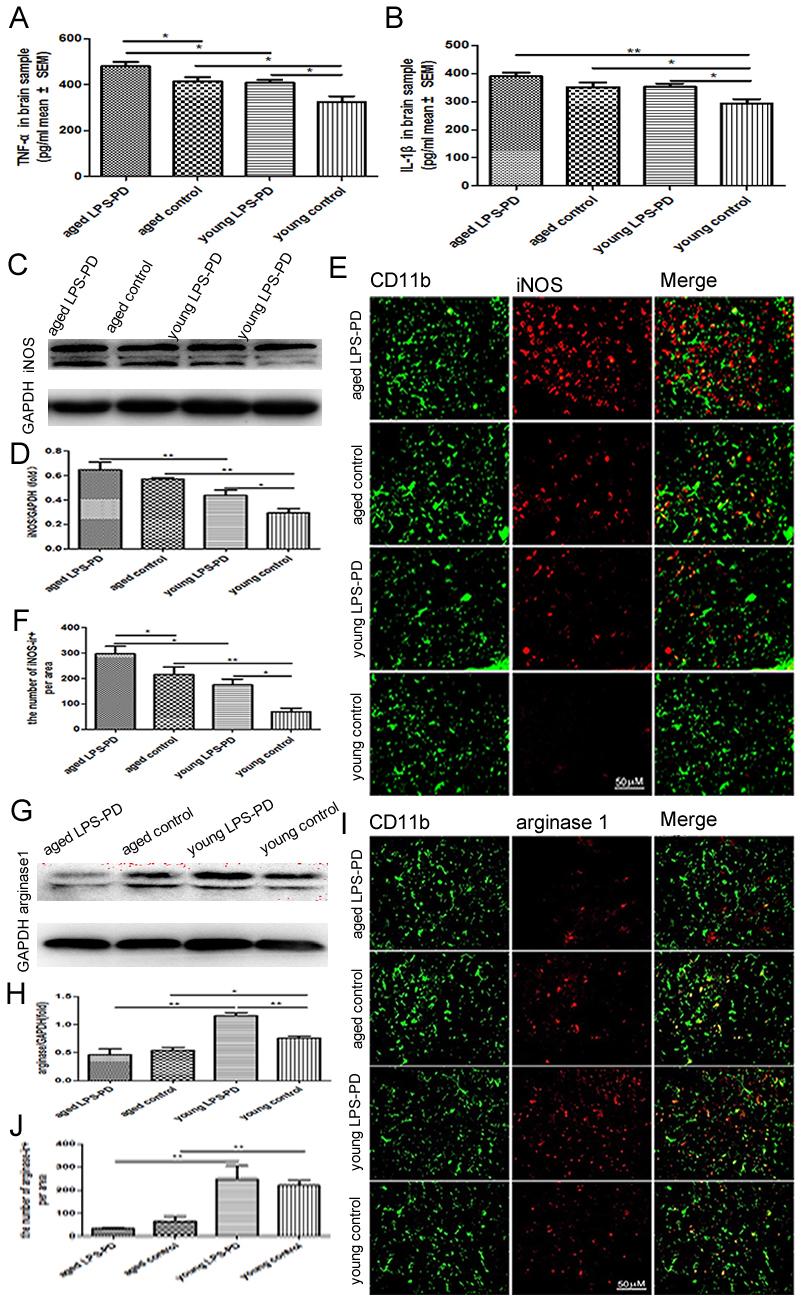
The levels of TNF-α, IL-1β, iNOS/Arg-1 expression, and iNOS/Arg-1 microglia in brain of young and aged mice stimulated with or without LPS Mice were dropped into the bilateral nasal cavity with saline or LPS in young or aged mice for 2 months. The levels of TNF-α and IL-1β in brain were assayed by ELISA. **A)** TNF-α; and **B)** IL-1β expression in brain was assayed by Western blot. **C)** Representative bands of iNOS immunostaining. **D)** Quantitative data of iNOS immunostaining. iNOS/Arg-1 positive microglia in brain was assayed by double immunohistochemistry of iNOS/Arg-1 and CD11b. **E)** Representative images of iNOS expression on CD11b^+^ microglia. **F)** Quantitative data of double positive cells. **G)** Representative bands of Arg-1 immunostaining. **H)** Quantitative data of Arg-1 immunostaining. **I)** Representative images of Arg-1 expression on CD11b^+^ microglia. **J)** Quantitative data of double positive cells. Quantitative results are mean ± SEM of 4 mice in each group. *p<0.05, **p<0.01.

Next, we measured the levels of pro-inflammatory of TNF-α and IL-1β in the homogenate of brain tissues by ELISA. Compared with young control group (TNF-α=325.9±23.9 pg/ml and IL-1β=293.4±15.3 pg/ml), the levels of TNF-α and IL-1β were increased significantly in aged control group (TNF-α=413.3±18.8 pg/ml and IL-1β=351.7±16.2 pg/ml, p<0.05 respectively) (Fig. 3A and 3B). The intranasal LPS instillation stimulated microglia to secrete TNF-α and IL-1β in young group (TNF-α=407.9±13.1 pg/ml and IL-1β=352.3±12.3 pg/ml, p<0.05 respectively) and aged group (TNF-α=480.3±18.7pg/ml, and IL-1β=391.2±13.0 pg/ml, p<0.05 respectively) (Fig. 3A and 3B).

### The polarization of M1 microglia with aging and LPS

Microglia are considered as a double-edged sword with both neurotoxic and neuroprotective effects and serve as the key component in neuroinflammation and neuroprotection of PD. iNOS and arg-1 are two most specific markers for M1 and M2 microglia, respectively. Consequently, we measured the expression of iNOS and arg-1 in the homogenate of brain tissues by western blot. The level of iNOS expression was elevated in aged mice (0.57±0.01) compared with young mice (0.29±0.04, p<0.01) (Fig. 3D). The intranasal LPS instillation significantly stimulated the induction of iNOS in young LPS-PD mice (0.44±0.05,p<0.05) and in aged LPS-PD mice (0.65±0.06) (Fig. 3D), although it did not reach statistical significance. The results of double immunofluorescence demonstrated that iNOS was expressed in CD11b positive microglia (Fig. 3E). The intensity of iNOS expression in different groups was basically consistent with the results from western blot (Fig. 3F).

However, the expression of arg-1 in young control group (0.76±0.04) was higher than in aged control group (0.54±0.05, p<0.05) (Fig. 3H). The intranasal LPS instillation significantly stimulated the expression of arg-1 in young LPS-PD mice (1.15±0.06,p<0.01), but did not influence the expression of arg-1 in aged LPS-PD mice (0.47±0.10,p>0.05) (Fig. 3H). The double immunofluorescence confirmed that arg-1 was expressed in CD11b positive microglia (Fig. 3I). The results were similar to those derived from western blot (Fig. 3J).

**Figure 4.**
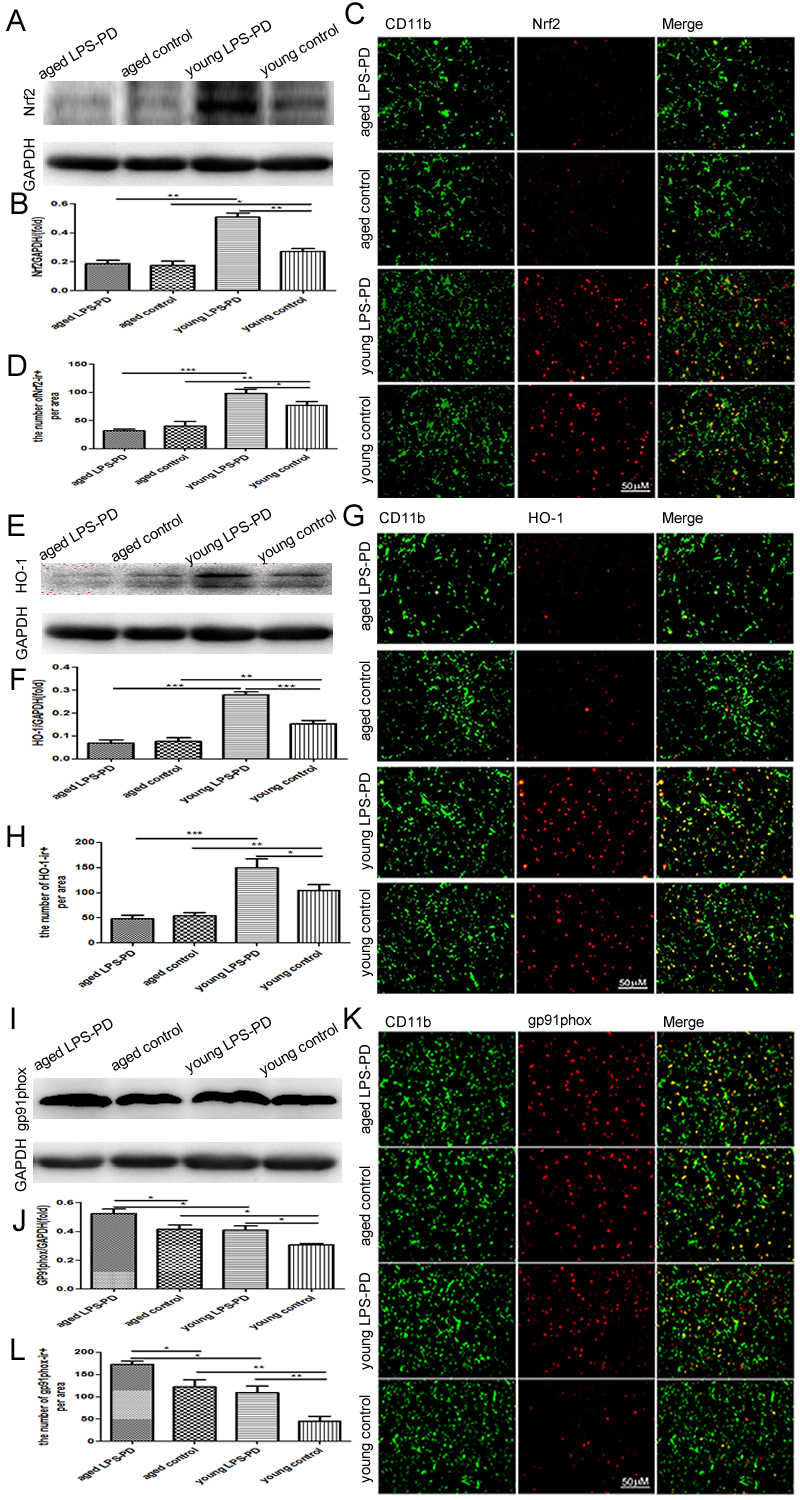
Nrf2/HO-1/Gp91phox expression and Nrf2/HO-1/Gp91phox microglia in brain of young and aged mice stimulated with or without LPS Mice were dropped into the bilateral nasal cavity with saline or LPS in young or aged mice for 2 months. Nrf2/HO-1/Gp91phox expression in brain was assayed by Western blot. **A)** Representative bands of Nrf2 immunostaining. **B)** Quantitative data of Nrf2 immunostaining. Nrf2/HO-1/Gp91phox positive microglia in brain was assayed by double immunohistochemistry of Nrf2/HO-1/Gp91phox and CD11b. **C)** Representative images of Nrf2 expression on CD11b^+^ microglia. **D)** Quantitative data of double positive cells. **E)** Representative bands of HO-1 immunostaining. **F)** quantitative data of HO-1 immunostaining; **G)** Representative images of HO-1 expression on CD11b^+^ microglia. **H)** Quantitative data of double positive cells; **I)** Representative bands of gp91phox immunostaining. **J)** Quantitative data of gp91phox immunostaining. **K)** Representative images of gp91phox expression on CD11b^+^ microglia.** L)** Quantitative data of double positive cells. Quantitative results are mean ± SEM of 4 mice in each group. *p<0.05, **p<0.01, ***p<0.001.

### Nrf2-HO-1 was declined with aging and increased with LPS in the young mice

A series of studies have demonstrated that neuro-inflammation and oxidative stress by microglia polarization may cooperate in the pathogenesis of PD. Nrf2 and its downstream effective molecule HO-1 is one of the important pathways of cell resistance to oxidative stress, anti-inflammatory response and cell protection. As shown in Figure 4, the expression of Nrf2 and HO-1 in young control group (Nrf2=0.27±0.02 and HO-1=0.15±0.01) was higher than in aged control group (Nrf2=0.18±0.03, p<0.05 and HO-1=0.08±0.02, p<0.01) (Fig. 4B and 4F). The intranasal LPS instillation strongly upregulated the expression of Nrf2 and HO-1 in young LPS-PD mice (Nrf2=0.51±0.03, p<0.01 and HO-1=0.28±0.01, p<0.001) (Fig. 4B and 4F), but did not influence the levels of Nrf2 and HO-1 in aged LPS-PD mice. The double immunofluorescence confirmed that Nrf2 and HO-1 were expressed in CD11b positive microglia (Fig. 4C and G). The results of Nrf2 and HO-1 were similar to those derived from western blot (Fig. 4D and 4H).

On the contrary, the expression of oxidase subunit gp91phox in young control group (0.31±0.01) was lower than in aged control group (0.42±0.03, p<0.05) (Fig. 4J). The intranasal LPS instillation upregulated the expression of gp91phox in young LPS-PD mice (0.41±0.03 p<0.05) and aged LPS-PD mice (0.52±0.03, p<0.05) (Fig. 4J). The double immunofluorescence confirmed that gp91phox was expressed in CD11b positive microglia (Fig. 4K). The results of gp91phox expression on CD11b microglia were similar to those derived from western blot (Fig. 4L).

## DISCUSSION

Aging usually is known as a series of time-dependent anatomical and physical alterations that exhibits the physiological and functional decline as well as homeostasis failure and eventually death [31-33]. Aging is supposed to be a stochastic process combining unpredictable and random events, which include primary aging determined by hereditary factors and secondary aging resulted from harmful factors in the environment [34-37]. Aging also is the largest risk factor for the sporadic PD according to the epidemiological studies during the development of PD [38, 39]. During the lifetime, native proteins are often exposed to stresses that can partially unfold and convert them into misfolded and aggregated status, which can in turn cause cellular damage and propagate to other cells [40, 41]. Especially in aged neurons, toxic aggregates may induce cell damage and death, ultimately leading to tissue degeneration via different mechanisms in PD. For unclear reasons, the cellular defense become gradually incapacitated with age, contributing to the onset of degenerative diseases. Understanding these mechanisms and the reasons for their incapacitation in late adulthood is key to design new therapies against the progression of neurodegenerative diseases. Our results suggest that with the increase of age, multiple balances are broken, formatting age-related first-strike; When environmental toxins, inflammatory/immune factors, oxidative stress are encountered again, two hits may accelerate the degeneration and loss of DA neurons.

This study demonstrates that the balance of cellular and molecular signals in aged mice are broken, and gradually transforms into harmful direction, as compared with young mice. This provides an opportunity to analyze what changes have taken place in cellular and molecular signals with age, and how they affect neurons. The results exhibited that Pole and ART behavioral tests were significantly prolonged in aged mice, and that nigral DA neurons were also remarkably declined as compared with young mice. In the elderly population without PD (mean age=88.5), nigral neuronal loss and α-synuclein immunopositive lewy bodies were observed. About 1/3 of cases had mild or more severe nigral neuronal loss and about 17% had lewy bodies. The average rate of nigral DA neuronl loss per decade was 4.7%-9.8% under the normal aging condition, while the number of neuronal cells in hippocampe, putamen, hypothalamus and meynert nucleus was relatively stable throughout the senility [42]. Therefore, nigral DA neurons are more easily lost because of aging, as compared to other areas related to other neurodegenerative diseases. The results in this study were consistent with previous studies [43]. Lieu et al found that older mice showed decreased motor performance in the pole test when compared to younger mice. Doxycycline-induced older mice displayed severe hind limb clasping and the most significant loss of dopamine in the striatum when compared to young and non-induced animals [44].

By far, the pathogenesis of PD could not be explained by a single mechanism, a variety of biological dysfunction, such as mitochondrial dysfunction, oxidative stress and neuroinflammation, may induce the loss of DA neurons, leading to the onset of PD when reach a certain threshold [45]. The question is what factors may affect the nigral DA neurons in the brain of aged mice. In this study, neuroinflammatory pathways were activated in the brain of aged mice. The expressions of TLR2, p-NF-kB/p65, TNF-α and IL-1β was significantly up-regulated as compared with young mice. Over the last decade, many studies have demonstrated that neuroinflammation is likely to contribute to nigrostriatal pathway degeneration[1, 46]. Although the role of inflammatory processes in modulating risk for development of PD has yet to be fully understood, prospective studies indicate that chronic use of NSAIDs reduced the incidence of PD [17, 19]. These findings raise the interesting possibility that environmental triggers may initiate cytokine-driven neuroinflammation and contribute to the development of PD in humans. Microglia adopt a more amoeboid morphology, characterized by larger cell bodies and shorter dendritic processes in aged brains compared to young microglia that show elaborate ramified processes and smaller cell bodies. These age-related microglia transformation from ramified to amoeboid morphology is characteristic of microglia activation by pro-inflammatory cytokines. Aged microglia are skewed toward a type 1 macrophage (M1) phenotype characterized by increased pro-inflammatory cytokine release, such as TNF-α and IL-1β [47]. This is also consistent with our findings that the polarization of aged macrophages was more likely to be M1 phenotype.

Nigrostriatal DA neurons may be uniquely vulnerable to neuroinflammatory insults that enhance cellular oxidative stress. In the current study, the expression of Nrf2 and HO-1, as antioxidant molecules, was remarkably declined, while the expression of gp91phox was elevated in aged mice compared with young mice, which is similar to the previous results [16]. The levels of Nrf2 protein in aged rats were lower than in the young rats [48]. Thus, aging is sufficient to diminish cardiac Nrf2-ARE binding activity, and aged Nrf2 knockout mice exhibited decreased expression of antioxidant target genes [49]. Disruption of Nrf2 signaling was reported in skeletal muscle from sedentary older humans and cardiac muscle from the aging rats. Aged mice showed similar loss in cellular redox capacity to those observed in Nrf2 knockout mice, suggesting that Nrf2 dysregulation with age may be responsible for the loss of cellular redox status. We speculate that the oxidative stress reaction was increased gradually, but the anti-oxidative stress function was decayed during the process of natural aging. With the increase of oxidative reaction and/or decrease of anti-oxidative ability, the destruction of oxidation-reduction homeostasis will cause neuronal damage and neurodegeneration.

Our previous studies displayed that intranasal instillation of LPS resulted in a progressive hypokinesia, selective loss of DA neurons, and reduction in striatal DA content, as well as α-synuclein aggregation in aged mice [28, 50]. In this study, intranasal instillation of LPS also induced PD model in both young and aged mice. Other studies showed that LPS resulted in a significant loss of DA neurons, accompanied by the activation of microglia and NF-kB pathway [51, 52]. LPS also induced the formation of α-synuclein fibrils with distinct structures, supporting the concept that LPS may generate unique synuclein fibril strains, which then spread to the CNS, leading to distinct pathological phenotypes and perhaps to different synuclein-related diseases [53]. Doxycycline can inhibit the degeneration of LPS-induced DA neurons. Its neuroprotective function is achieved by downregulating the microglia MHC II expression [54]. Compared with young mice, the behavioral abnormalities and DA neuron loss were more serious in aged mice, which may be related to the severity of inflammatory response and oxidative stress in the brain of aged mice. Chronic inflammatory and oxidative stress could cause some damage to DA neurons in aged rats. Given LPS stimulation, further activation of microglia should amplify the inflammatory response and oxidative stress in the brain. It is possible to aggravate the loss of DA neurons, exacerbating the behavioral deficits of PD. Our data support the concept that continuous exposure to a pro-inflammatory environment drives exaggerated changes in the production and release of inflammatory mediators and oxidative stress that interact with age to result in the impairment of DA neurons.

LPS usually triggers a release of pro-inflammatory mediators from microglia/macrophages through a TLR4/NF-κB signaling pathway [55]. It is well known that LPS stimulates the upregulation of Nrf2 and HO-1 [56-59]. Interestingly, the expression of Nrf2 and HO-1 was upregulated by LPS only in young LPS-PD mice, but not in aged mice, suggesting that the upregulation of Nrf2 and HO-1 is associated with the age, independently of TLR2-p-NF-kB/p65 status. It has been reported that aging is tightly associated with redox events [60]. The young Nrf2 knockout mice displayed lower expression of detoxifying enzymes but not antioxidant enzymes, but old Nrf2 knockout mice showed no differences in either detoxifying or antioxidant enzymes [61]. Treatment with anetholetrithione caused Nrf2 to accumulate significantly in cells from young animals, but not old, indicating a lack of new Nrf2 synthesis in cells from aged animals [62]. In addition, Nrf2 activation and the induction of Nrf2-target genes were lost in the middle-aged mice (21 months), compared with that of young adults (6 months) [63]. These results show that the antioxidant capacity was reduced in aged mice and could not be increased by LPS stimulation. In young mice, LPS not only stimulated microglia to produce inflammatory molecules and oxidative stress, but also induced antioxidant molecules, which achieves dynamic balance between oxidation and anti-oxidation, and inhibits the inflammatory response through Nrf2 signaling pathway, revealing that young mice have strong compensation ability.

### Conclusion

Aging is the most important risk factor of PD due to chronic neuroinflammation and oxidative stress microenvironment in the brain. Under the micro-environment, DA neurons suffer from persistent injury during the aging. When hit by microenvironmental risk factors again, neuronal death may be accelerated. Compared with aged mice, the young mice may have a strong ability to compensate the harmful micro-environment mediated with environmental toxins. In reality, there are several questions remaining to be answered. What mechanisms control the loss in response to oxidative stress of the Nrf2/HO-1 signaling in old mice? To what degree should the second hit of the environment factors be fatal in the aged mice? Finally, how many of these effects of aging will also be important components of aging phenotype in humans?
